# Efficiency of Automated Viral RNA Purification for Pediatric Studies of Dengue and Zika in Hyperendemic Areas

**DOI:** 10.1155/2023/1576481

**Published:** 2023-09-30

**Authors:** Sandra L. Delgado, Piedad M. Perilla, Doris M. Salgado, María Clemencia Rojas, Carlos F. Narváez

**Affiliations:** ^1^División de Inmunología, Programa de Medicina, Facultad de Salud, Universidad Surcolombiana, Neiva, Huila, Colombia; ^2^Área de Pediatría, Universidad Surcolombiana, Neiva, Huila, Colombia; ^3^Laboratorio de Salud Pública, Secretaría de Salud Departamental del Huila, Neiva, Huila, Colombia

## Abstract

The isolation of nucleic acids is a critical and limiting step for molecular assays, which prompted the arrival in Colombia of automated purification instruments during the SARS-CoV-2 pandemic. The local application of this technology in the study of tropical diseases, such as dengue and zika, is beginning to be tested. We evaluated the efficiency of the automated extraction of viral RNA for studies of pediatric dengue and zika. Clinical samples of children with dengue that were well characterized through RNA isolation by silica columns and serotype-specific nested RT-PCR (DENV-1 *n* = 7, DENV-2 *n* = 5, and negatives *n* = 8) in addition to 40 pediatric plasma samples spiked with ZIKV (strain PRVA BC59) and 209 from negative pre-epidemic children were analyzed. RNA from patients was extracted by two automated standard and high-throughput protocols on the KingFisher™ Flex instrument. The isolated RNA was evaluated for concentration and purity by spectrophotometry, for structural and functional integrity by electrophoresis and expression of the RNase P gene, and usefulness in serotype-specific DENV detection by conventional and real-time RT-PCR. For the evaluation of ZIKV RNA, the commercial TaqMan Triplex® assay was used, along with a well-tested in-house RT-qPCR assay. The concentration of RNA (5.2 vs. 7.5 ng/*μ*L, *P*=0.03) and the number of integral bands (9 vs. 11) were higher with the high-throughput protocol. However, the number of specimens serotyped for DENV by RT-qPCR was comparable for both protocols. The cycle thresholds of the TaqMan Triplex® commercial kit and the in-house assay for the detection of plasma ZIKV RNA isolated with the standard protocol showed a strong association (*r* = 0.93, *P* < 0.0001) and a Cohen Kappa index of 0.98 when all 249 samples were analyzed. These preliminary results suggest that automated instruments could be used in studies of cocirculating flaviviruses that have represented a public health problem in recent decades in Colombia. They boast advantages such as efficiency, precision, time savings, and lower risk of cross-contamination.

## 1. Introduction

The infection by the severe acute respiratory syndrome coronavirus 2 (SARS-CoV-2) emerged as one of the greatest challenges to modern public health. Since its description in December 2019, SARS-CoV-2 infection has caused hundreds of millions of infections and millions of deaths, in addition to a devastating impact on the global economy [1]. A consequence of the SARS-CoV-2 pandemic was the visibility of tremendous technological and infrastructural inequalities on top of the public health problem imposed by the pandemic in some regions of Colombia, such as the low availability of modern diagnosis instruments and shortage of personnel with training to perform viral genome detection and sequencing assays [2]. Diagnostic tests and viral phylogeny studies are aspects that the World Health Organization deems critical axes for monitoring COVID-19 [3]. Both the public and private sectors made significant investments in real-time polymerase chain reaction (qPCR) instruments and automated devices for the extraction of nucleic acids from bodily fluids. In Colombia, for example, in the first half of 2020, the Ministry of Science called for strengthening the capacities of science and technology to address emerging and re-emerging diseases such as COVID-19, adding an investment of approximately USD 50 million from the “Sistema General de Regalías” (SGR), a situation never seen in the country (https://www.minciencias.gov.co/convocatorias/plan-bienal-convocatorias-fctei/convocatoria-del-fondo-ctei-del-sgr-para-el).

Dengue and zika are emerging and re-emerging viral diseases that have affected tropical areas for years without any of the measures taken have stopped the growth in cases, especially dengue in the last decade. In fact, in 2019, the Americas suffered a large dengue virus (DENV) epidemic, with more than 3,100,000 cases of symptomatic infections and 1,770 fatal cases, 90% in children [4]. Colombia was no exception, as the country recorded a total of 127,500 cases, including 1,400 severe dengue cases (https://www3.paho.org/data/index.php/es/temas/indicadores-dengue/dengue-nacional/9-dengue-pais-ano.html). Currently, another large DENV epidemic is occurring, and up to August of 2023, more than 3 million symptomatic infections have been registered in the Americas.

The efficient and adequate extraction of RNA is a critical and limiting factor for the diagnosis and study of viral infections, and many of the failures of downstream molecular studies are due to problems in RNA purification [5]. RNA is highly susceptible to enzymatic degradation [6, 7]. Classically, solid-phase RNA extraction can be performed manually through the use of conjugated columns and centrifugation or using magnetic microspheres [8]. However, the application of these procedures has disadvantages, such as their high costs, time-consuming protocols, risks of contamination with genomic DNA, and limited numbers of specimens that can be assayed per round [9]. To solve this, automated purification instruments have come to market. Automated instrument offers other advantages such as the high number of samples performed for round and the lower risk of human mistakes. One of these instruments is the KingFisher™ Flex (Thermo Fisher), a portable platform for the automated extraction of RNA, DNA, and proteins, which can perform 24 or 96 isolates per cycle [10]. During the SARS-CoV-2 pandemic, this equipment has been widely used in the diagnosis of respiratory infection from nasal or oropharyngeal swab samples with extraction protocols adapted for this type of biological fluid [11, 12].

The efficiency of this technology in the study of DENV and ZIKV infection in children has not been proven and their performance will have a high impact on public health. Here, we evaluate the efficiency of automated RNA extraction applied to the identification of DENV and ZIKV in pediatric natural infection and spiked experiments, respectively.

## 2. Methods

### 2.1. Patients and Samples

Twenty plasma samples that had been collected from febrile pediatric patients (younger than 14 years old), and were previously characterized to have confirmed or rejected DENV infection by both a conventional nested RT-PCR [13] and ELISA for NS1 (NS1 Panbio™ Early ELISA, cat: DEN02P), were used to measure the efficiency of automated RNA purification. They were characterized as DENV-1 (*n* = 7), DENV-2 (*n* = 5), and DENV-negative infections (*n* = 8). To perform serotype-specific DENV nested RT-PCR, RNA isolated by silica columns and by centrifugation through the QIAamp® Viral RNA mini-Kit (Qiagen, cat: 52904, Hilden, Germany) was used, following the manufacturer's instructions. The manually purified RNA had a median (range) concentration of 42.9 ng/*µ*L (9.4–496) and a *λ*260/*λ*280 nm ratio of 3.2 (1.6–3.9), as evaluated by spectrophotometry.

**Figure 1:**
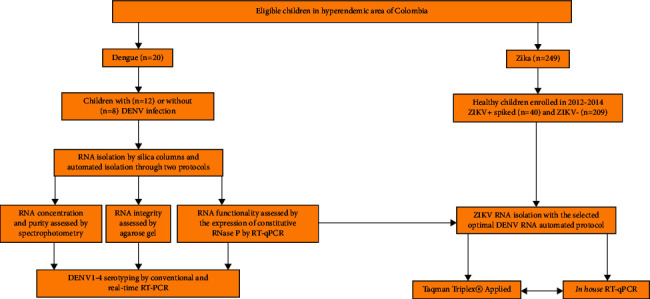
Flow chart of the study. Children were enrolled in Huila, a department located in southern Colombia.

For the ZIKV analyses, automated RNA isolation was performed on 249 plasma samples from healthy pediatric patients taken between 2012 and 2014 (before ZIKV had come to the Americas). For the spiked experiments, 40 samples were added to *in vitro* known amounts of ZIKV strain PRVA BC59 previously titrated by a focus-forming assay [14] in a range of 50–100,000 focus-forming units (FFU)/mL. The study design is presented in Figure 1.

This study followed all the ethical standards in research in the Helsinki Declaration and was approved by the Ethics Committee of the Facultad de Salud of Universidad Surcolombiana and the Ethics, Bioethics, and Research Committee of the Hospital Universitario de Neiva (Approval code 24-04).

### 2.2. Automated RNA Purification

For RNA purification, the commercial MagMax™ Viral/Pathogen II kit (Applied Biosystems, catalog: A48383) and the automated purification instrument KingFisher™ Flex (Thermo Scientific™, catalog: 5400630, Singapore) were used. Two purification protocols programmed in the instrument were evaluated. The first one was called MVP_FLEX, henceforth called “high-throughput” [15]. Briefly, 5 *µ*L of proteinase K was placed in each well of the extraction plate, and 200 *µ*l of plasma was added. Then, 275 *µ*l of the extraction mixture containing binding buffer (265 *µ*l) and the magnetic beads (10 *µ*l) were added. In an automated manner, the equipment agitated the plate for 5 minutes and performed a first wash with 500 *µ*l of wash buffer, followed by 500 *µ*l of 80% ethanol and a final wash with 250 *µ*l of 80% ethanol. After drying the beads for 5 minutes at 72°C, the purified RNA was collected in 50 *µ*l of elution buffer. The high-throughput protocol is recommended for the isolation of RNA from plasma. The second protocol tested was MVP_2Wash_200_Flex, hereafter called “standard.” Unlike MVP_FLEX, the standard protocol has no last washing step with 80% ethanol. This modification reduces the amount of supplies and reagents and shortens the extraction time of 96 clinical specimens to 25 minutes from the beginning of the mounting of the plate in the equipment. During the COVID-19 pandemic, this protocol was recommended by the manufacturer for the isolation of RNA from nasopharyngeal swabs preserved in a viral transport medium for the diagnosis of SARS-CoV-2 by RT-qPCR [15].

The automated isolates included culture supernatants from Vero-76 cells that had been mock-infected (negative control) or a mix of supernatants of Vero-76 cells infected with DENV-1 or DENV-2 serotypes (positive control). In some experiments, ultrapure water was included as a no-template control (NTC). The purified RNA was immediately used to perform the assays or, in some cases, frozen at −80°C.

### 2.3. Quantification of Purified RNA

The concentration and purity of the isolated RNA were evaluated by spectrophotometry with a NanoDrop 2000 (Thermo Scientific, Wilmington, DE, USA) following the manufacturer's instructions. To establish the purity, the absorption ratio of the RNA was obtained at wavelengths of 260 and 280 nm (*λ* 260/280 nm), as previously reported [16].

### 2.4. Integrity Evaluation in Agarose Gel

The appearance of the total purified RNA migrating in an electrophoretic field was used to evaluate the integrity of the RNA. A 2% agarose gel was prepared, and 5 *µ*l of ethidium bromide was added. Ten microliters of purified RNA plus 1 *µ*l of loading buffer was added to each lane of the gel. The RNA migrated for 1 h at a voltage of 110 V. Finally, the gel was analyzed with the Universal Hood II imager, GelDoc™ XR system (Bio-Rad).

### 2.5. Evaluation of the Expression of the RNase P Gene

To evaluate the presence and quality of the purified RNA, the constitutive expression of the ribonuclease P (RNase P) gene was measured. For this, specific primers, and probes (F-AGATTTGGACCTGCGAGCG; R-GAGCGGCTGTCTCCACAAGT; and probe HEX-TTCTGACCTGAAGGCTCTGCGCG-BHQ1) [17], were prepared at a concentration of 10 *µ*M. Amplification was performed using the SuperScript™ III Platinum™ One-Step qRT-PCR System (Invitrogen, cat: 11732-088; Lot: 2223827; Invitrogen Life Technologies. Carlsbad. CA 92008. USA), following the manufacturer's recommendations. Of this mixture, 20 *µ*L was placed in each well. Subsequently, 5 *µ*L of the purified RNA was added, obtaining a final volume of 25 *µ*L. The following controls were included in the plate: NTC, mix Vero-76 cells DENV-1- and DENV-2-infected (positive control), and mock-infected (negative control). The plate was amplified in the QuantStudio™ 5 thermocycler (Thermo Applied Biosystems, cat: A28134) and the amplification was performed with the following thermal profile: 15 min at 50°C, 2 min at 95°C, 15 sec at 94°C, and finally 30 sec at 58°C, the last two repeating 42 cycles.

### 2.6. Serotype-Specific Identification of DENV by Conventional and Real-Time RT-PCR

The utility of purified RNA for the detection of DENV-1 and DENV-2 was tested by conventional RT-PCR and RT-qPCR. For the first case, a widely used conventional nested RT-PCR was used [13]. To detect DENV by RT-qPCR, the following serotype-specific sequences were used in a single not multiplex format: DENV-1 F 5′ TGA TGA ACA ACC AAC GRA AAA A; DENV-1 R 5′ GTT TCT CCC GCG TTT CAG CAT; DENV-1 probe 5′ CGG STC GAC CGT CTT TC FAM; DENV-2 F 5′ CTG CAR GGA CGA GGA CCA TT; DENV-2 R GGG ATT GTT AGG AAA CGA AGG A; DENV-2 probe 5′ AAA CTG TTC ATG GCC CTG GTG GCR FAM. Ten microliters of the mix was added to the plate, and 2.5 *µ*L of the isolated viral RNA was added. The assembly was enhanced on the Eco™ Illumina or QuantStudio™ 5 instruments with the following thermal profile: 20 min at 50°C; 2 min at 95°C; and 40 cycles of 15 seconds at 95°C and 1 minute at 60°C. In all experiments, a mixture of culture supernatant from Vero-76 cells infected with each of the DENV serotypes or supernatant from uninfected cells was used as a positive and negative control, respectively.

### 2.7. ZIKV Spiked Assay

Spike experiments were used to detect ZIKV in human plasma specimens. The supernatant of Vero-76 cells (ATCC, cat: CRL-1587) infected by ZIKV strain PRVA BC59 was titrated by focus formation assays [14], then added in different amounts to the plasma of 40 healthy pediatric volunteers taken before the arrival of ZIKV to the Americas. The range of FFU tested was 50–100,000 FFU/mL, all with a final constant volume of 200 *µ*L that was used for purification with the automated protocol. To corroborate the specificity of the automated isolation, 209 plasma samples from healthy volunteers taken before 2015, to whom ZIKV was not added *in vitro*, were also included for automated RNA purification.

### 2.8. Detection of ZIKV by RT-qPCR

For the detection of ZIKV, the commercial TaqMan™ Zika Virus Triplex® Kit (Applied Biosystems, cat: A31746), kindly gift from ARC Análisis de Colombia S.A.S, was used as gold standard following the manufacturer's instructions. This commercial multiplex RT-qPCR kit is widely used for the identification of arboviruses. This assay detects DENV, ZIKV, and CHIKV arboviruses in a single well. To detect ZIKV by a second RT-qPCR assay, we followed a previously described and widely used test [18].

### 2.9. Statistical Analysis

Statistical analysis was performed with GraphPad Prism version 7 software (GraphPad Software Inc., San Diego, CA, USA). Nonparametric statistical tests were run, and medians and ranges were reported. For the analysis of two independent groups, the Mann‒Whitney test was used. As a measure of association, the association coefficient (*r*, Pearson test) was calculated. The Cohen Kappa index was performed to compare the adjusted agreement of the detection among molecular methods. *P* < 0.05 was considered significant in all analyses.

## 3. Results

### 3.1. Characteristics of the Patients Included

Twenty patients with suspected DENV infection, clinically classified as dengue without warning signs according to the revised WHO classification of 2009, were included (Table 1). For the detection of ZIKV, spiked experiments were performed in 40 pre-epidemic specimens out of the 249 included.

As shown in Table 1, there was no gender or age predominance among any of the groups. Patients with DENV infection were in the febrile phase of the disease with a median (range) time of fever of 3 days (1–5). Confirmed DENV infections corresponded to 7 for DENV-1 and 5 due to DENV-2. There were no DENV infections in 8 cases.

### 3.2. Concentration and Purity of the RNA Isolated Automated with Magnetic Microspheres

**Figure 2:**
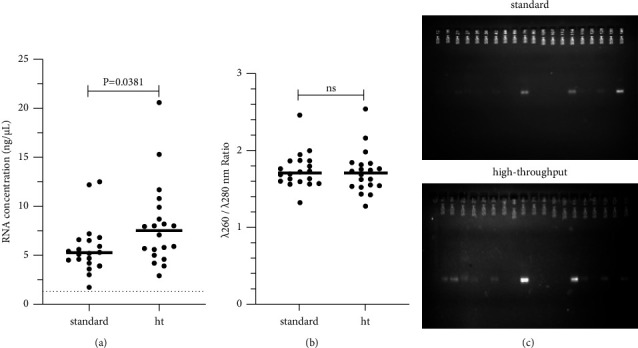
Characteristics of RNA isolated automatically. (a) Absolute concentration of RNA automatically purified from the plasma of pediatric patients with and without confirmed DENV infection. (b) Relative purity of the samples analyzed by the *λ* ratio 260/280. *λ*260/*λ*280 nm: ratio of the maximum absorbance obtained at the wavelengths (*λ*) of 260 and 280 nm. The detection limit of the NanoDrop™ 2000 = 1.3 ng/*µ*L is shown as a dotted line. The *P* value obtained with the Mann‒Whitney test is shown for each case. (c) Electrophoresis in 2% agarose gel of the total RNA purified automatically from plasma using the standard protocol (upper) and the high-throughput protocol (lower). The results of 20 pediatric samples are shown.

Initially, the concentration and purity of the isolated RNA were evaluated with the two automated purification protocols on the 20 samples confirmed or ruled out of DENV infection. As shown in Figure 2(a), the RNA isolated with the high-throughput extraction protocol had a 30% higher concentration than that isolated with the standard protocol (*P*=0.0381, Mann‒Whitney test). The purities obtained with the two automated protocols were generally acceptable (median 260/280 nm ratio >1.71) and similar to each other, regardless of the protocol used (*P* > 0.9, Mann‒Whitney test) (Figure 2(b)).

The structural integrity of the RNA isolated from each of the 20 clinical samples was evaluated through electrophoresis in a 2% agarose gel.  Figure 2(c) shows an image of the gels. The presence of complete RNA bands was observed in the gels with the two extraction protocols evaluated. The identifiable number of bands with the standard protocol was 9/20, while that of the high-throughput protocol was 11/20 (*P*=0.3761, Fisher's exact test). The intensity of the bands present was regularly higher with the use of the high-throughput protocol compared to the standard (Figure 2(c)).

### 3.3. Functional Analysis of the Expression of the RNase P Constitutive Gene in the Isolated RNA

**Figure 3:**
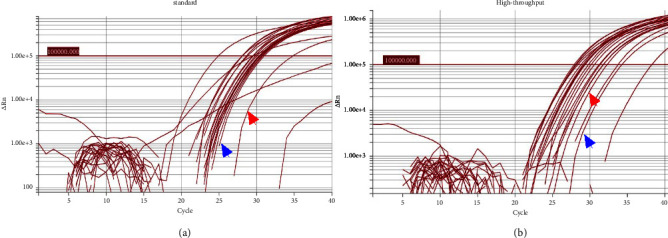
Amplification by RT-qPCR of the RNase P gene in automatically purified RNA by the standard protocol (a) and the high-throughput protocol (b). The cycle threshold (*C*_*t*_) value was previously established at ΔRn 1 × 10^5^ by prior validations. The arrows blue and red show the amplification curves of the RNase P gene in positive and negative control, respectively. Results of 20 pediatric plasma samples added to positive and negative control (cell culture supernatant with or without DENV infection) are shown.

To test the presence and functionality of the RNA isolated automatically from clinical pediatric samples, we analyzed the expression of the RNase P gene. As shown in Figure 3, the presence of RNA was noted in most specimens regardless of the extraction protocol used. Of the 20 specimens evaluated, RNase P amplification was detected in 18 with the standard and the high-throughput protocol (Figure 3). Additional analyses showed that there were no differences in the expression level of the RNase P gene between the RNA purified by the two protocols as evaluated by comparison of cycle threshold (*C*_*t*_) among both groups (Supplemental Figure 1). An interesting result was that the RNA extracted from the culture supernatant of Vero-76 cells infected (positive control) or not (mock) with DENV also showed amplification of the RNase P gene (Figure 3). To confirm this finding, we isolated automatically RNA from 1 × 10^6^ human peripheral blood mononuclear cells (PBMC) and Vero-76 cells and analyzed the RNase P through RT-qPCR with the same primers and probe sequences. As shown in Supplemental Figure 2, although in much lesser extension than human PBMC, amplification was detected in uninfected Vero-76 in the highest RNA concentration tested.

In summary, with automated purification, was noted an efficient amplification of a widely used human constitutive gene to evaluate the presence and quality of purified RNA.

### 3.4. DENV Serotype-Specific Amplification of RNA from Automatically Purified Clinical Samples

**Figure 4:**
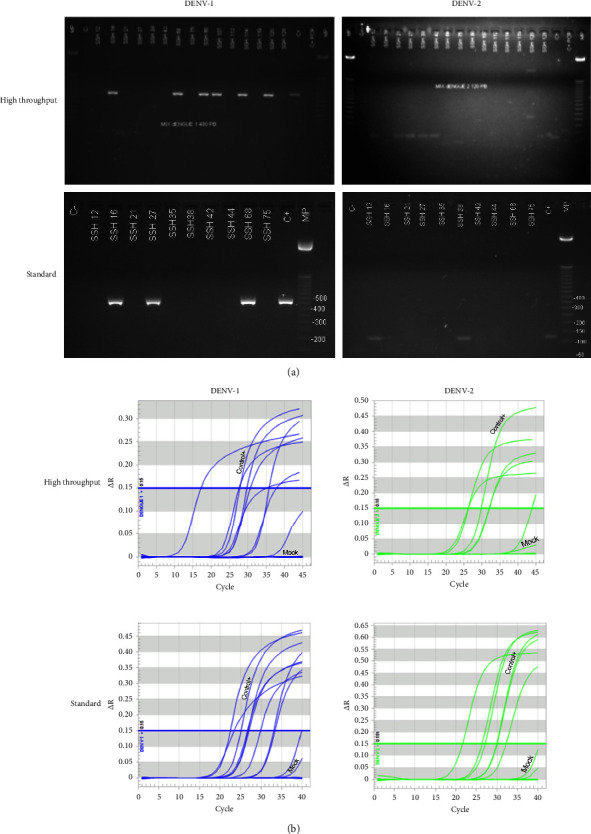
Use of the RNA purified automatically in the serotype-specific detection assays for DENV. (a) Detection of DENV-1 (left panel, molecular weight 482 bp) and DENV-2 (right panel, molecular weight 119 bp) by conventional RT-PCR on clinical plasma samples from 12 pediatric patients with DENV infection (DENV-1 = 7 and DENV-2 = 5) and 8 negatives for DENV infection. RNA was isolated with the high-throughput protocol (upper) and standard (down). The figure shows four negative samples (for high-throughput) in the upper images and 5 positives (for standard protocol) in the lower images, as other samples were run in a separate gel. C+: supernatant mixture of Vero-76 cells infected with each of the DENV serotypes. C−: mock. (b) Amplification curves of DENV-1 (in blue) and DENV-2 (in green) by RT-qPCR on automatically purified RNA from the plasma of naturally infected patients using the high-throughput (upper) and the standard (down) protocols. The curve generated by the respective cell cultured DENV (control+) and a mock of each condition is shown. The value taken as positive corresponded to a sample with a *C*_*t*_ ≤ 38.

tThe most important test of the functionality of isolated RNA was its usefulness for the detection of serotype-specific DENV from clinical specimens by RT-PCR. Therefore, conventional RT-PCR was initially performed for serotype-specific detection from the RNA isolated by both protocols. With the high-throughput protocol, amplification was noted in 11 of the 20 samples analyzed (Figure 4(a)), with six infections reported by DENV-1 and five by DENV-2, which was highly concordant with the conventional RT-PCR run on purified RNA with silica columns, used as the gold standard. The conventional RT-PCR using the RNA purified with the standard protocol showed amplification in 7 specimens (DENV-1 = 5 and DENV-2 = 2, Figure 4(a)).

Next, the 20 clinical specimens were also subjected to an RT-qPCR assay that evaluated each of the serotypes. Positive and negative controls worked as expected. Of the 20 clinical specimens analyzed by serotype-specific RT-qPCR for DENV after the two automated extraction protocols, most cases with confirmed infection were identified regardless of the extraction protocol used (Figure 4(b)). Serotype-specific amplification was noted in 11/20 analyzed specimens with the high-throughput protocol, while with the standard protocol, amplification was noted in 13/20.  Table 2 summarizes the results of the RT-qPCR assays for DENV obtained with the two automated isolation protocols compared to the gold standard, which was the conventional nested RT-PCR performed on RNA isolated with silica membrane chemistry. As shown in Table 2, of the 20 clinical specimens analyzed, 17 were concordant between the three methods (conventional RT-PCR and the RT-qPCR performed on RNA extracted by the two automated protocols). In two cases (SSH 38 and SSH 80), the standard automated extraction protocol, but not the high-throughput protocol, was consistent with the result shown by nested RT-PCR. In one other sample (SSH 75), the RT-qPCRs of the two automated extraction protocols were consistent with each other, but not with the nested RT-PCR performed on RNA extracted with a silica column (Table 2). The comparison of the two automated isolation protocols has an agreement of 0.9 (lower limit 0.66, upper limit 0.98, Cohen Kappa index: 0.79) when the serotype-specific RT-qPCR for DENV was applied. However, in the 2 out to 3 mismatch cases, the standard but not the high-throughput protocol was concordant with the DENV nested RT-PCR with the RNA isolated with silica column (Table 2).

In summary, our results show that the automated extraction of RNA from the plasma of clinical specimens of pediatric patients with confirmed natural DENV infection using the KingFisher™ Flex instrument is useful for downstream molecular studies. Because the standard protocol was at least comparable for the detection of DENV by RT-qPCR than the high-throughput protocol (Table 2), added to its lower use of reagents and shorter extraction duration, this automated protocol was selected for subsequent experiments with RT-qPCR for ZIKV.

### 3.5. Efficiency of Automated RNA Isolation in the Identification of ZIKV in Children Plasma

To extend the previous findings to ZIKV, which is another flavivirus important in human health and currently cocirculates with DENV in Colombia, spiked experiments were designed. This was due to the low availability of naturally infected febrile specimens. Known quantities of ZIKV strain PRVA BC59 that had been titrated by a focus formation assay [14] were added to plasma from healthy children taken between 2012 and 2014 before ZIKV was seen in Colombia. The RNA was extracted with the KingFisher™ Flex instrument using the standard protocol evaluated above for DENV. The presence of the genome of this flavivirus in the purified RNA was simultaneously evaluated by the TaqMan® Triplex ZIKV Kit (Applied Biosystems) and a previously reported and widely used RT-qPCR assay [18].

Amplification of the constitutive controls of the TaqMan® Triplex (cyclophilin A, PPIA gene) and in-house (RNase P gene) assays demonstrated the presence and good quality of RNA after automated isolation in tested specimens (Supplemental Figure 3).

As shown in Table 3, no false positives and only one false negative were found in all the samples analyzed (Table 3), when the commercially available kit was the gold standard. An agreement and Cohen Kappa index of 0.99 and 0.98 were obtained, with positive and negative predictive values of 100% and 99%, respectively.

**Figure 5:**
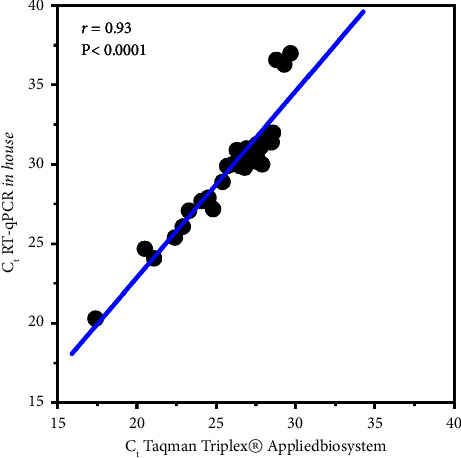
Measurement of the relationship (Pearson association coefficient (*r*)) between the *C*_*t*_ for detection of ZIKV in the spiked specimens tested by the gold standard assay (TaqMan™ Zika Virus Triplex® kit, Applied Biosystems) and the well-tested in-house method described by Lanciotti et al. [18]. The graph shows the *r* and its respective *P* value from the analysis of 40 spiked samples.

Although the fluorochromes of the probes used to detect ZIKV in the commercial and the in-house assays were different, we determined whether there was a relationship between the *C*_*t*_ obtained by the two RT-qPCR assays in the samples positive for ZIKV. As shown in Figure 5, a high coefficient of determination (*r* = 0.93, *P* < 0.0001) was found, suggesting that the two methods identified the same relative amount of viral RNA.

In summary, the RNA isolated automatically with the KingFisher™ Flex instrument allowed us to reliably detect different amounts of ZIKV particles in pediatric plasma.

## 4. Discussion

The purity and quality of RNA is a key factor in the performance of molecular assays. Different purification methods based on RNA chemistry and the use of a solid phase, such as guanidinium thiocyanate-phenol-chloroform [19], silica matrices, and magnetic microspheres [20], have been described. Their performance depends on multiple factors, such as the type and quantity of the source sample [8, 21]. In this work, we compared the concentration and purity of RNA isolated from the plasma of pediatric patients naturally infected by DENV using silica columns and automated isolation based on magnetic spheres. We tested two versions of the latter methods called standard and high-throughput, which are recommended for RNA purification from nasopharyngeal swabs and plasma, respectively [15]. These isolation methods (silica and magnetic beads) were evaluated because they are two of the most used and most efficient methods for the isolation of RNA from fluids and tissues currently available [21].

The amount of RNA expected in plasma is lower than that expected when the original specimen is solid tissue or whole blood. The concentration obtained with the automated extraction of RNA from plasma was generally comparable to that reported with chemical methods, such as TRIzol-based isolation [22]. A higher concentration of purified RNA was obtained with the high-throughput protocol (approximately 30% higher) than the standard protocol. Thus, changes in the number of washes with agents such as ethanol can modify the amount of RNA isolated from the plasma of clinical samples, since the concentration of contaminating factors that affect the efficiency of purification, such as proteins and lipids, is higher in plasma samples than in other biological fluids conserved in buffers [23]. Despite the increase in the concentration of the RNA obtained, the increase in the number of ethanol washes of the high-throughput protocol did not increase the purity of the RNA, as evaluated by the *λ*260/280 nm ratio, and this purity was in accordance with that obtained in a recent study that used the same KingFisher™ Flex automated extraction instrument and commercial purification kit for purification [24].

High fragility and easy degradation, even during the purification process, are critical characteristics of the RNA [25]. Therefore, we evaluated the structural integrity of the RNA purified by the two automated protocols employing gel electrophoresis of the total purified RNA. In general, strong unique bands were observed in the electrophoretic gels with RNA from both automated protocols, confirming the integrity of the RNA, and although more bands were observed in the high-throughput group, a result possibly related to the higher concentration of RNA obtained by this protocol.

The expression of human RNase P was measured to further evaluate the quality and suitability of the isolated RNA for subsequent molecular assays. RNase P is an endoribonuclease that has a single-copy gene in each human cell, with constitutive and relatively constant expression levels of the gene [26]. Therefore, it has been widely used as an endogenous control of molecular diagnostic assays of RT-qPCR, especially during the COVID-19 pandemic [27]. The RNA purified with the two automated protocols from the plasma of febrile patients with or without DENV infection was functional, since in nearly all the specimens, there was an amplification of the RNase P gene. Of note, late amplification of the human RNase P gene was also noted in the RNA isolated from Vero-76 cell culture supernatants and this finding was extended with additional experiments. This result disagrees with previous reports where the expression of human RNase P was analyzed in Vero cells infected with SARS-CoV-2 using the same primers/probe sequences as used here [28]. RNase P is a conserved protein even between different kingdoms [29]. However, the highly variable concentration of pure total RNA obtained from Vero cell culture supernatants, the different nucleic acids isolation methods, and the interaction between fluorochromes labeling the probes will partially explain this result.

The RNA isolated automatically from clinical samples of DENV infection was used for the serotype-specific detection of DENV-1 and DENV-2 by conventional and real-time RT-PCR. The KingFisher™ Flex instrument has been used to isolate RNA in studies of the interaction of flaviviruses in hosts such as mosquitoes and humans [30–33] and even in studies of Chikungunya virus [34, 35], although few studies have compared in-depth the efficiency of this automated method and its application to molecular methods in humans [24]. In general, the purified RNAs were useful for viral serotyping by the types of PCR used, although with some subtle differences. Comparison with conventional RT-PCR on RNA purified with silica columns and centrifugation was consistent with previous studies [24]. Real-time serotyping using RNA isolated with the KingFisher™ Flex instrument was concordant in at least 86% of cases, with an advantage for the standard protocol of 2/20 additional concordant samples over the high-throughput protocol. Although the high-throughput protocol was superior in factors such as the concentration of isolated RNA, the number of RNA bands observed in the agarose gel, and number of serotypes identified by conventional RT-PCR, the performance of the standard protocol in serotype-specific RT-qPCR for DENV was comparable.

A strong and significant relationship was found between the *C*_*t*_ for ZIKV obtained with the two RT-qPCR methods that we compared on the purified RNA, supporting and extending the usefulness of automated isolation for the amplification of target sequences within the ZIKV genome by different platforms. The agreement of the RT-qPCR assay with the commercial kit was high. The range of detection of spiked ZIKV RT-qPCR experiments showed comparable results with the low number of circulating infecting viral particles reported in naturally infected patients [36], although samples isolated from naturally infected children were not analyzed here and it constitutes an important limitation of the study. Other important limitations included the low number of children with dengue enrolled, the use of only one instrument for the automated RNA isolation, and the noninclusion of DENV-3 and DENV-4 infected clinical samples.

Thus, with this work, we demonstrated the potential utility of the automated instrument for RNA isolation for molecular studies of cocirculating arbovirus in pediatric settings.

## 5. Conclusion

The automated isolation of RNA allowed the identification of DENV in a serotype-specific manner, in addition to ZIKV in an efficient manner. Evidence is presented of the potential use of automated instruments applied after the SARS-CoV-2 pandemic to other cocirculating viruses that have represented a serious problem to the public health of Colombia in recent decades.

## Figures and Tables

**Table 1:** Characteristics of the patients included in the study.

	Dengue suspected children *n* = 20	Plasma samples from healthy children experimentally infected or not with Zika *n* = 249		*P*
		Spiked positive *n* = 40	Negative *n* = 209	
Gender (F/M)	10/10	24/16	83/126	0.323^a^
Age (years)	11 (1–14)	8 (1–13)	10 (1–14)	0.09^b^
Days of fever	3 (1–5)	N/A	N/A	N/A
DENV-1^*∗*^	7	—	ND	ND
DENV-2^*∗*^	5	—	ND	ND
DENV Negative^*∗*^	8	40	ND	ND

^a^Fisher's exact test was used. ^b^Kruskal–Wallis test was applied. N/A: not applicable. ND: not done. ^*∗*^RT-PCR positive and negative samples were also positive and negative for circulating NS1 as evaluated by ELISA.

**Table 2:** Summary of results of detection of DENV obtained from purified RNA isolated automated with standard and high-throughput protocols.

Code	Silica column-based isolation QIAGEN	Automated purification KingFisher^TM^ Flex		Result
		Standard	High-throughput	
	RT-PCR^a^	RT-qPCR^b^		
SSH 12	DENV-2	DENV-2	DENV-2	Matched
SSH 16	DENV-1	DENV-1	DENV-1	Matched
SSH 21	DENV-2	DENV-2	DENV-2	Matched
SSH 27	DENV-2	DENV-2	DENV-2	Matched
SSH 35	NEG	NEG	NEG	Matched
SSH 38	DENV-2	DENV-2	NEG	Mismatched
SSH 42	DENV-2	DENV-2	DENV-2	Matched
SSH 44	NEG	NEG	NEG	Matched
SSH 68	DENV-1	DENV-1	DENV-1	Matched
SSH 75	NEG	DENV-1	DENV-1	Mismatched
SSH 80	DENV-1	DENV-1	NEG	Mismatched
SSH 105	NEG	NEG	NEG	Matched
SSH 107	DENV-1	DENV-1	DENV-1	Matched
SSH 112	NEG	NEG	NEG	Matched
SSH 114	DENV-1	DENV-1	DENV-1	Matched
SSH 119	NEG	NEG	NEG	Matched
SSH 120	DENV-1	DENV-1	DENV-1	Matched
SSH 128	NEG	NEG	NEG	Matched
SSH 130	NEG	NEG	NEG	Matched
SSH 146	DENV-1	DENV-1	DENV-1	Matched
Control + ^c^	DENV-1, DENV-2	DENV-1, DENV-2	DENV-1, DENV-2	Matched
Mock^d^	NEG	NEG	NEG	Matched

^a^Conventional nested RT-PCR was used as a gold standard^13^. ^b^Serotype-specific RT-qPCR used to detect DENV-1 and DENV-2. ^c^Mix of supernatants of Vero-76 cell culture infected with DENV-1 and DENV-2. ^d^Supernatants of Vero-76 cells uninfected with DENV.

**Table 3:** Performance of the automated purified RNA to detect ZIKV evaluated through the 2 × 2 table for the two RT-qPCR assays.

	TaqMan Triplex® ZIKV Applied Biosystems		Total
	Negative	Positive	
*In house* negative	209	1	210
*In house* positive	0	39	39

Total	209	40	249

## Data Availability

All relevant data from this study were included in the figures and the supplemental information. If required, additional information can be solicited from the corresponding author.

## Supplementary Materials

Supplementary Materials Supplemental Figure 1: RNase P amplification from RNA isolated automatically. RNA was purified with two protocols with the KingFisher^TM^ Flex instrument and the expression of the constitutive gene was evaluated by RT-qPCR. The median is shown. Ct: cycle threshold, ns: nonsignificant, and HT: high-throughput. Supplemental Figure 2: RNA from 1 × 106 human peripheral blood mononuclear cells (PBMC) and Vero-76 cells infected or not with ZIKV were purified automatically. Dilutions in Log_10_ were realized. Amplification of the human RNase P was detected by RT-qPCR. Red line: RNase P curves. Blue line: ZIKV curves. NTC: no-template control. Ct ≤37 was accepted as positive. Supplemental Figure 3: internal control for amplification of constitutive cyclophilin A and RNase P genes. RNA from pediatric plasma spiked for ZIKV (*n* = 40) was isolated automatically with the standard protocol. A commercial kit (left plot) and in-house (right plot) RT-qPCR assays for ZIKV detection were tested. NTC: no-template control highlighted with the black arrowheads.
