# Supplementary material for: Efficiency of Automated Viral RNA Purification for Pediatric Studies of Dengue and Zika in Hyperendemic Areas
Source: J Trop Med. 2023 Sep 30;2023:1576481. doi: 10.1155/2023/1576481 (PMC10560119; doi:10.1155/2023/1576481)
Supplement: Supplementary Materials — Supplemental Figure 1: RNase P amplification from RNA isolated automatically. RNA was purified with two protocols with the KingFisherTM Flex instrument and the expression of the constitutive gene was evaluated by RT-qPCR. The median is shown. Ct: cycle threshold, ns: nonsignificant, and HT: high-throughput. Supplemental Figure 2: RNA from 1 × 10^6^ human peripheral blood mononuclear cells (PBMC) and Vero-76 cells infected or not with ZIKV were purified automatically. Dilutions in Log10 were realized. Amplification of the human RNase P was detected by RT-qPCR. Red line: RNase P curves. Blue line: ZIKV curves. NTC: no-template control. Ct ≤37 was accepted as positive. Supplemental Figure 3: internal control for amplification of constitutive cyclophilin A and RNase P genes. RNA from pediatric plasma spiked for ZIKV (n = 40) was isolated automatically with the standard protocol. A commercial kit (left plot) and in-house (right plot) RT-qPCR assays for ZIKV detection were tested. NTC: no-template control highlighted with the black arrowheads. [file 1576481.f1.pdf]

Supplemental Information

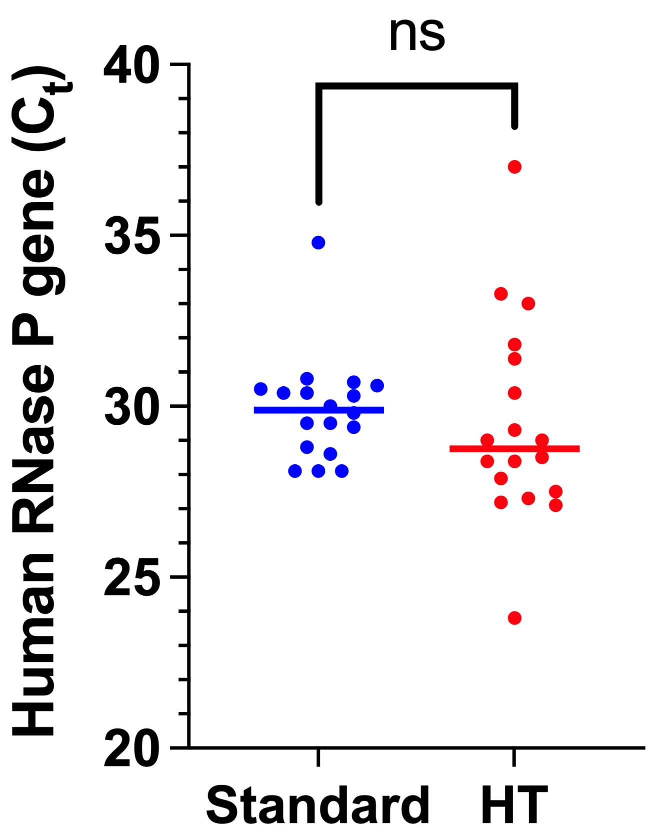

Supplemental Figure 1. RNase P amplification from RNA isolated automatedly. RNA was purified with two protocols with the KingFisher™ Flex instrument and the expression of the constitutive gene was evaluated by RT-qPCR. The median is shown. C_t: Cycle threshold. ns: non-significant. HT: high-throughput.

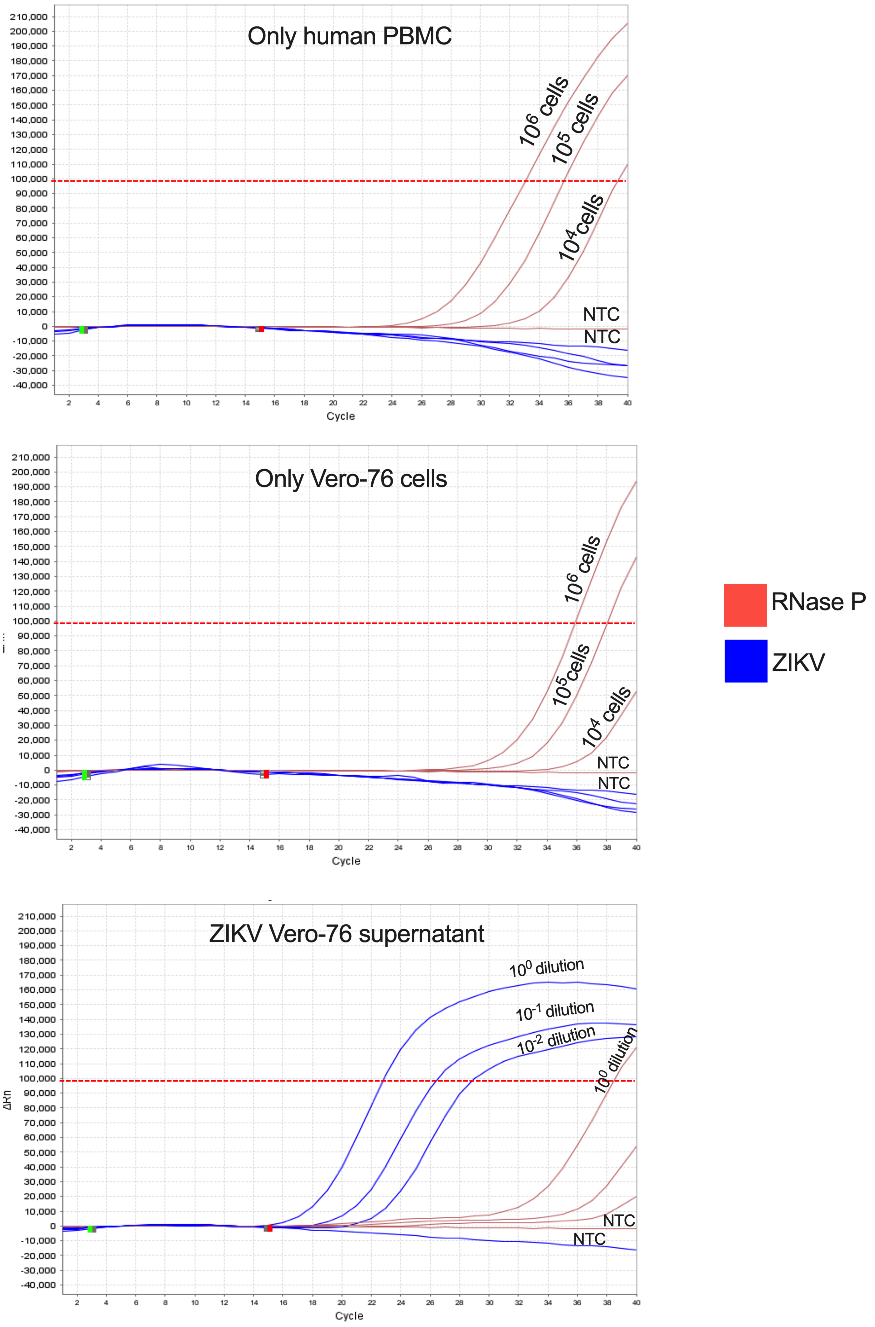

Supplemental Figure 2. RNA from $1x10^6$ human peripheral blood mononuclear cells (PBMC) and Vero-76 cells infected or not with ZIKV were purified automatedly. Dilutions in Log_{10} were realized. Amplification of the human RNase P was detected by RT-qPCR. Red line: RNase P curves. Blue line: ZIKV curves. NTC: no-template control. $C_t \leq 37$ was accepted as positive.

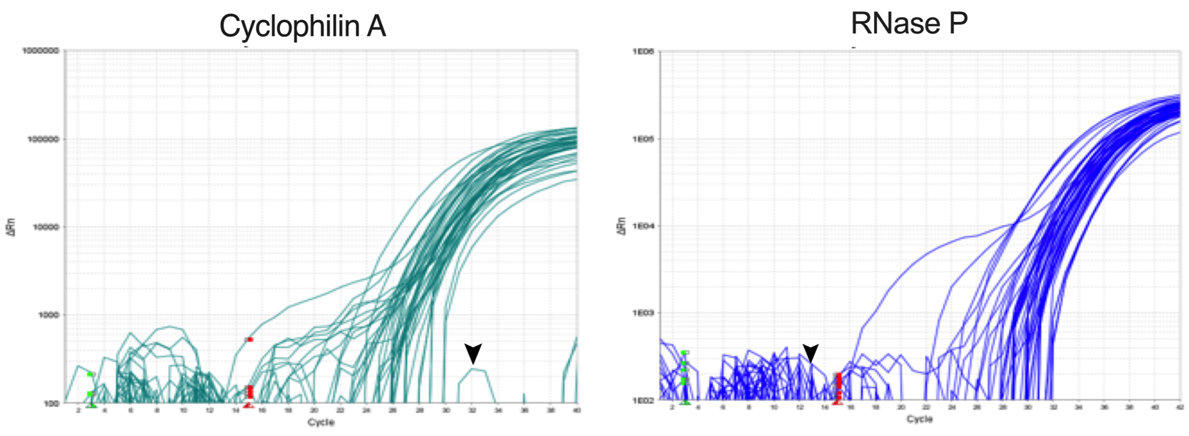

Supplemental Figure 3. Internal control for amplification of constitutive Cyclophilin A and RNase P genes. RNA from pediatric plasma spiked for ZIKV (n=40) was isolated automatedly with the standard protocol. A commercial kit (left plot) and in-house (right plot) RT-qPCR assays for ZIKV detection were tested. NTC: no-template control is highlighted with the black arrowheads.
